# Development of the Alcohol Use Disorders Identification Test-Consumption (AUDIT-C) Score as a Predictor of Alcohol Withdrawal Syndrome in Trauma Patients at a Community Level 1 Trauma Center

**DOI:** 10.7759/cureus.103164

**Published:** 2026-02-07

**Authors:** Melinda Bottenfield, Karleigh Curfman, Shirin Siddiqi, Thomas Simunich, Avi Robinson, Russell Dumire, Shawna Morrissey

**Affiliations:** 1 Surgery, Conemaugh Memorial Medical Center, Johnstown, USA; 2 Research and Performance Excellence and Quality, Conemaugh Memorial Medical Center, Johnstown, USA

**Keywords:** alcohol screening, alcohol use disorder (aud), alcohol withdrawal prediction, alcohol withdrawal syndrome, audit-c, ethanol intoxication, geriatric trauma

## Abstract

Background and objective

The Clinical Institute Withdrawal Assessment for Alcohol-Revised (CIWA-Ar) is an assessment tool that guides symptom-triggered therapy (STT) in alcohol withdrawal syndrome (AWS) patients. Institutionally, CIWA-Ar is used for STT when patients admit to daily alcohol use or arrive intoxicated. Given the lack of validated screening tools for predicting AWS, we hypothesized that CIWA-Ar and STT were used inefficiently, causing poor resource stewardship and overtreatment. Our current protocol is to complete the Alcohol Use Disorders Identification Test-Consumption (AUDIT-C), an evidence-based screening tool for hazardous alcohol use. Given this protocol and the absence of verified screening tools for alcohol withdrawal prediction, we aimed to analyze AUDIT-C efficacy in predicting AWS to guide STT.

Methods

A retrospective review was performed of admission AUDIT-C responses between January 1, 2018, and December 31, 2018. Given the vague documentation of AWS diagnosis, an alcohol withdrawal syndrome score (AWS Score) was created based on current literature and was statistically confirmed. Per our criteria, AWS was defined as an AWS score of ≥ 3 in patients with moderate alcohol use.

Results

The study population included 662 trauma patients, predominantly geriatric (age ≥ 65 years, 68%) and female (60%). In the setting of moderate alcohol use, AUDIT-C was a statistically significant predictor for AWS (logistic regression model, χ2(1) = 172.371, p < 0.0005), with a 90.0% sensitivity, 96.2% specificity, positive predictive value of 52.9%, and negative predictive value of 99.5%. To provide clinicians a guide for more objective utilization of alcohol withdrawal protocols, an AUDIT-C threshold of ≥ 5 was identified using binary logistic regression and receiver-operating characteristic curve (ROC) analyses.

Conclusion

We noted AUDIT-C scores of ≥ 5 at the time of admission in hospitalized trauma patients with moderate alcohol use to predict AWS. Given these findings, we propose that AUDIT-C scores may be reliable guides for implementing alcohol withdrawal protocols for the treatment of this patient population.

## Introduction

Alcohol use and abuse are extremely prevalent problems in current society, for which approximately 48% to 50% of trauma patients are found to be at risk for hazardous drinking habits or have evidence of an alcohol use disorder (AUD) [1-3]. Nearly 50% of trauma patients are found to have detectable serum levels of alcohol upon hospital admission [4], with 10% of those experiencing a readmission within one year to the same hospital for a new traumatic injury [4]. Although documentation of AUD is robust in trauma patients, the rates of developing alcohol withdrawal syndrome (AWS) in this population, fortunately, remain low (0.8% to 0.9%) [1,5]. Despite the low rate, AWS remains a prime topic of interest in trauma research due to its risk of significant morbidities and possible effects on future related traumatic events. Several published studies have documented an association between trauma patients with AWS and an increased length of hospital stay, higher readmission rates, more frequent need for mechanical ventilation, and diagnosis of pneumonia [1,6,7]. Interestingly, AWS symptoms have also been found to be more pronounced and of longer duration in geriatric patients, increasing their delirium risk [8-10].

Despite the significant prevalence of alcohol use in adult trauma patients, an obvious lack of validated AWS screening tools persists [11]. Instead, various methods, such as patient admission blood alcohol concentration (BAC) or reports of hazardous habits on screenings, have been haphazardly used to place patients on Clinical Institute Withdrawal Assessment for Alcohol-Revised (CIWA-Ar) protocols to guide symptom-triggered therapy (STT). The CIWA-Ar is a 10-question assessment applied to those at moderate risk for alcohol withdrawal and validated for AWS symptom identification and classification of severity [12,13]. It is not copyrighted and may be reproduced freely.

For CIWA-Ar to be used effectively, patients must first be screened for increased risk of AWS [14], which represents a major application flaw, as currently there are no applicable evidence-based screening tools validated in the prediction of alcohol withdrawal. For this reason, there is concern that CIWA-Ar protocols are potentially being misused, which can lead to the overuse of recommended sedatives in STT [14,15]. One study at the Mayo Clinic revealed that only 48% of admitted patients were appropriately assessed and correctly placed on CIWA-Ar [14]. This is especially concerning in both trauma and elderly patients who are inherently predisposed to hospital delirium, medication-induced over-sedation, and respiratory depression [16]. 

The 10-question Alcohol Use Disorders Test (AUDIT) has been validated for the accurate detection of hazardous alcohol use in patient populations [17-19]. This assessment has been further abbreviated into three surveys: AUDIT-Consumption (C), AUDIT-Dependence (D), and AUDIT-Picinelli (P) (see Appendix A). The AUDIT-C survey is composed of the first three questions of the AUDIT questionnaire, which assess the quantity and frequency of alcohol consumption [20,21]. Next, the AUDIT-D is comprised of the three subsequent elements, which determine the degree of alcohol dependence. Finally, the AUDIT-P represents the remaining four constituents, assessing for psychosocial problems related to alcohol use. Generally, the higher the score, the more likely it is that a person's drinking is affecting his or her safety. However, the AUDIT questionnaire has not been validated in the prediction of AWS. The AUDIT-C/D/P is available for use in the public domain.

Several studies have been published that analyze these different components of the AUDIT questionnaire and their clinical application. For example, four individual studies demonstrated that a higher AUDIT-C score is reflective of severe alcohol misuse and was associated with an increased risk of trauma-related hospitalizations, fractures, and postoperative complications [22-25]. Literature published by Pecoraro et al. studied the application of AUDIT-PC, a combination of AUDIT-P and AUDIT-C, in the prediction of alcohol withdrawal in hospitalized patients. This report demonstrated that an admission AUDIT-PC score ≥ 4 was associated with 91.0% sensitivity and 89.7% specificity for AWS and could be used as a reliable tool in withdrawal prediction [11]. Given their success in validating the use of AUDIT-PC for AWS prediction in hospitalized patients, we decided to perform a similar study at our institution. Of note, the Pecoraro et al. [11] study was not performed specifically with an increased-risk trauma population, nor did it individually evaluate the AUDIT-C and AUDIT-P components of the AUDIT-PC score.

Our study evaluated the use of AUDIT-C alone in predicting AWS in hospitalized adult trauma patients. Despite other resources, like the Christiana Care study [26], which evaluated different AUDIT components or combinations thereof, the AUDIT-C was specifically used in our study for several reasons. First, as previously described, a higher AUDIT-C score suggests severe alcohol use, which is associated with an increased risk for trauma-related medical care and complications; therefore, we felt that the AUDIT-C would be most applicable to a trauma patient-focused study [22-25]. Next, per the guidelines of the American College of Surgery Verification Review Committee for level 1 trauma center accreditation, routine screening for hazardous alcohol use is a requirement [27]. As such, the AUDIT-C as a screening tool had been implemented at our institution before the commencement of this study and was available for retrospective review. Finally, the brevity of the AUDIT-C, containing only three questions as opposed to the 10-question AUDIT or the seven-question AUDIT-PC, lends itself to efficient screening in busy emergency departments [17].

Additional studies have demonstrated that independently, a higher AUDIT-C score is reflective of severe alcohol misuse and was associated with an increased risk of trauma-related hospitalizations, fractures, and postoperative complications [22-25]. Therefore, we hypothesized that the AUDIT-C specifically would be significantly predictive of AWS in hospitalized trauma patients. Additionally, we expected this to be associated with a discrete AUDIT-C score that would inform more appropriate application of CIWA-Ar and STT protocols. Finally, as this study aimed to fill a previously identified void in validated and reliable tools predictive of inpatient development of AWS, we predicted that insights gained from assessing our application of the AUDIT-C would reveal opportunities for more selective use of alcohol withdrawal protocols.

This work was previously presented virtually as an abstract and as a poster at the American Association for the Surgery of Trauma conference in 2020.

## Materials and methods

This study was approved by the Institutional Review Board (IRB) of the Conemaugh Memorial Medical Center (approval no. 19-25). This retrospective cohort analysis included adult patients (age ≥ 18 years) admitted to the trauma service at a community level 1 trauma center from January 1, 2018, through December 31, 2018, regardless of traumatic mechanism or injury (n = 1,379).

**Figure 1:**
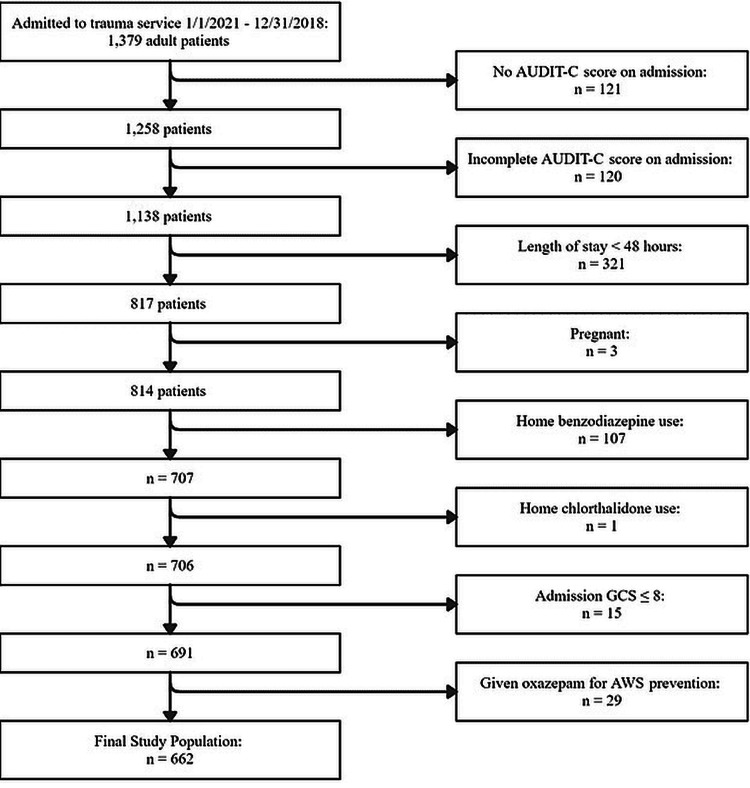
Study population exclusion process The data presented represent patient counts. AUDIT-C: Alcohol Use Disorders Identification Test-Consumption, AWS: Alcohol withdrawal syndrome

Patients were excluded for the following reasons: length of stay < 48 hours (n = 321), lack of documented complete AUDIT-C score on admission (n = 256, which includes 15 cases in which the clinical state was unclear, pregnancy (n = 3), home benzodiazepine use (n = 107), home chlorthalidone use (n = 1), and patients given oxazepam as a fixed dose taper to prevent AWS during their hospitalization (n = 29). After exclusions, the final study population consisted of 662 patients (Figure 1). 

Data was extracted from both the electronic medical record (EMR) and trauma database. Values at admission for Glasgow Coma Scale score (GCS), vital signs, blood alcohol content (BAC), and AUDIT-C scores (total and individual components) were collected. Additionally, documented alcohol use, CIWA-Ar score, inpatient administration of benzodiazepines (lorazepam, diazepam, or oxazepam), use of soft-limb restraints, and hospital length of stay were obtained. Patient history, specifically home medications as listed in the EMR at admission, was used to identify comorbidities that could potentially confound AWS determination, including anxiety, dementia, seizure disorders, and Parkinson’s disease.

Defining alcohol withdrawal syndrome via novel AWS score

Given the absence of a documented diagnosis of AWS in the EMR and that most patients were not placed on our institution’s CIWA-Ar protocol for AWS identification, a generalized method based on the collected patient data was retrospectively devised to identify AWS or the risk for its occurrence. The AWS Score was formed from the Diagnostic & Statistical Manual of Mental Disorders (DSM)-V criteria for the diagnosis of AWS [28], known risk factors of AWS and/or symptoms reflective of the autonomic hyperactivity seen in AWS, insights from CIWA-Ar methodology, and AUDIT-C scoring that implies 'moderate' alcohol use. As complete documentation of all factors contained within the DSM-V proved to be elusive, use of the DSM-V AWS definition alone was not feasible. As with the DSM-V AWS criteria, our study AWS score was based upon the patient having exhibited a history of 'moderate' alcohol use [28]. However, the DSM-V criteria do not specify the quantity or duration of alcohol use that would be considered moderate. Therefore, we defined 'moderate' alcohol use as an admission BAC of greater than 0.08%, the legal limit for intoxication in the United States of America [29], and/or an admission AUDIT-C (see Appendix A) of greater than or equal to three (female) and four (male), which defines 'hazardous drinking use' [20,30]. The AUDIT-C is available for use in the public domain. 

Initially, our AWS score included sub-scores of CIWA-Ar. However, as most patients in this study were not placed on CIWA-Ar protocol (n = 609) and, as such, were not regularly assessed for CIWA-Ar-monitored symptoms such as tremors, insomnia, anxiety, or hallucinations, the inclusion of CIWA-Ar subscores was abandoned. Additionally, the incorporation of CIWA-Ar sub-scores allowed for too much selection bias towards patients on CIWA-Ar protocols. Despite these difficulties, a total CIWA-Ar score greater than 10 was used as an AWS score component since that is reflective of complex alcohol withdrawal (see Appendix B) [12,13]. The CIWA-Ar is not copyrighted and may be reproduced freely. As CIWA-Ar monitored symptomology was lacking, surrogate variables were needed. To that end, the necessity of limb restraints was chosen as a surrogate marker for agitation, as soft limb restraints were often used in patients showing signs of severe agitation and jeopardizing the safety of themselves and others. Furthermore, the use of benzodiazepines in naïve patients was used as a surrogate for anxiety, agitation, or insomnia. 

A GCS score of less than 15 was used to reflect confusion. To reduce confounding by the concomitant presence of dementia or Parkinson’s disease, where the patient may function at a lower baseline GCS, the score threshold was lowered to less than 14. Abnormal vital signs that reflected autonomic hyperactivity consistent with an increased risk of AWS were also included [12]. Minimal values were set at a higher-than-normal threshold for our study to minimize secondarily inflated scores in patients who may have experienced abnormal vital signs due to pain and trauma. 

To ensure that a higher AWS score was associated with heavier alcohol use, the component questions of the AUDIT-C score were also included [20]. The threshold score of the first question, assessing frequency of drinking, was set to 4 as a reflection of daily alcohol use. The threshold score of the second question, quantity of drinking, was set to greater than 1, correlating with consuming more than four drinks in one sitting. Finally, the third question, analyzing the frequency of binge drinking (defined as 6 or more drinks in one sitting), was given a threshold of 2 or greater, consistent with binge drinking at least two to three times per week. 

Both AWS scores and documented CIWA-Ar scores were adjusted for patient comorbidities to minimize potential score inflation and confounders. Parkinson’s disease (patients prescribed carbidopa-levodopa with or without entacapone) nullified CIWA-Ar sub-scores of confusion and tremors. Dementia (designated in our study as patients prescribed donepezil or memantine) nullified CIWA-Ar sub-scores of confusion. Seizure disorders (patients prescribed divalproex, temazepam, carbamazepine, or phenobarbital) nullified CIWA-Ar sub-scores of tremors. Anxiety/sleep disorders (patients prescribed quetiapine, melatonin, or haloperidol) nullified CIWA-Ar sub-scores of anxiety. Our AWS scoring system is detailed in Table 1.

**Table 1:** The AWS score The AWS score presented here was developed for this study. AWS: Alcohol withdrawal syndrome, GCS: Glasgow Coma Scale, AUDIT-C: Alcohol Use Disorders Identification Test-Consumption, CIWA-Ar: Clinical Institute Withdrawal Assessment for Alcohol-Revised

Criteria			Explanation of use
A. History of alcohol use:			
A-1		BAC > 0.08	U.S. legal criteria for alcohol intoxication
A-2		AUDIT-C Score ≥ 3 (female) and ≥ 4 (male)	Definition of hazardous drinking use
B. Each item counted as 1 point:			
B-1		AUDIT-C: frequency of drinking score = 4	Equivalent to daily alcohol use
B-2		AUDIT-C: # of drinks at a time score > 1	Equivalent to 3-4+ drinks at a time
B-3		AUDIT-C: frequency of 6+ drinks in one sitting score > 2	Equivalent to weekly binge-drinking
B-4		CIWA-Ar Score > 10	Reflective of complex AWS
B-5		Benzodiazepine given during admission	Surrogate for anxiety, agitation, or insomnia
B-6		Soft limb restraints used	Surrogate for severe agitation
B-7		GCS score < 15;	Surrogate for confusion
---		If dementia or Parkinson’s disease, the criterion is lowered to GCS < 14	
B-8		Heart rate (HR) > 109 beats per minute (bpm)	Symptoms reflective of the autonomic hyperactivity seen in alcohol withdrawal syndrome
B-9		Systolic blood pressure (SBP) > 179 mmHg	
B-10		Respiratory rate (RR) > 24 breaths per minute (BRPM)	
B-11		Temperature (T) > 38.3 °C	
Item B-4 was adjusted for comorbid conditions likely to confound scoring, e.g., dementia, Parkinson's disease, anxiety, seizure disorders			
Scoring (0-12)			
Both Part A and Part B must meet or exceed the individual scoring threshold for that part in order to proceed to the calculation of an alcohol withdrawal syndrome score.			
Part A threshold:			
Either item A-1 or A-2 must be true			
Part B threshold:			
11 items, each scored as 1 point			
If both elements of Part A are true, add 1 point to the Part B score before comparing the Part B sum to its threshold			
When the Part B score is ≥ 3, the threshold for this part has been met			
When the thresholds for both Parts A & B have been met, the AWS score is calculated as: 1 + the sum of Part B			
An AWS score ≥ 3 is associated with alcohol withdrawal syndrome symptomology			
An AWS score ≤ 2 implies that the symptoms are unlikely to be caused by alcohol withdrawal syndrome			

Statistical analysis

The AWS score was confirmed using binary logistic regression and receiver-operating characteristic curve (ROC) analyses. For this study, AWS was defined as an AWS score ≥ 3 in the setting of documented/known moderate alcohol use. The SPSS Statistics version 24 was used to conduct the statistical analyses (IBM Corp., Armonk, NY, USA). The overall type I error (alpha) was set at 0.05. 

## Results

Of the 662 patients admitted to the trauma service between January 1, 2018, and December 31, 2018, who met selection criteria, 30 (4.5%) patients experienced alcohol withdrawal, as defined by an AWS score of ≥ 3. The population in this study was found to be predominantly female (59.6%) with a mean age of 70.6 years old. Further demographic information regarding the study population can be seen in Table 2. The frequency distribution of AUDIT-C scores is detailed in Table 3. 

**Table 2:** Demographics Continuous variable (age) has been represented as mean (range) and all other variables as a count and percentage (%). AUDIT-C: Alcohol Use Disorders Identification Test-Consumption, BAC: Blood alcohol concentration

Characteristics	Total study population	Subgroup of population with AUDIT-C ≥ 5
			(n = 662)	(n = 51)
	Mean age, years (range)	70.6 (18-106)	52.0 (19-81)
Women, n (%)	395 (59.6)	12 (23.5)
Men, n (%)	267 (40.4)	39 (76.7)
Use of restraints, n (%)	51 (7.7)	8 (15.7)
Anxiety/sleep disorder, n (%)	47 (7.1)	7 (13.7)
Dementia, n (%)	43 (6.5)	0
Admission BAC > 0.08, n (%)	33 (5.0)	24 (47.1)
Benzodiazepines given, n (%)	20 (3.0)	9 (17.6)
Seizure disorder, n (%)	20 (3.0)	1 (2.0)
Parkinson’s disease, n (%)	12 (1.8)	0

**Table 3:** Frequency distribution of AUDIT-C scores The data has been presented as a count and the percentage (%) of the total study population represented by the count. Total study population is n = 662 (100). AUDIT-C: Alcohol Use Disorders Identification Test-Consumption

AUDIT-C Score	Portion of Total Study Population per Each Possible Audit-C Score	Portion of Total Study Population per Audit-C Score of 0, (1-5, inclusive), (5-12, inclusive)
0	495 (74.7)	495 (74.5)
1	60 (9.1)	116 (17.5)
2	22 (3.3)	
3	15 (2.3)	
4	19 (2.9)	
5	15 (2.3)	51 (7.7)
6	7 (1.1)	
7	4 (0.6)	
8	5 (0.7)	
9	5 (0.7)	
10	7 (1.1)	
11	3 (0.5)	
12	5 (0.7)	

**Figure 2:**
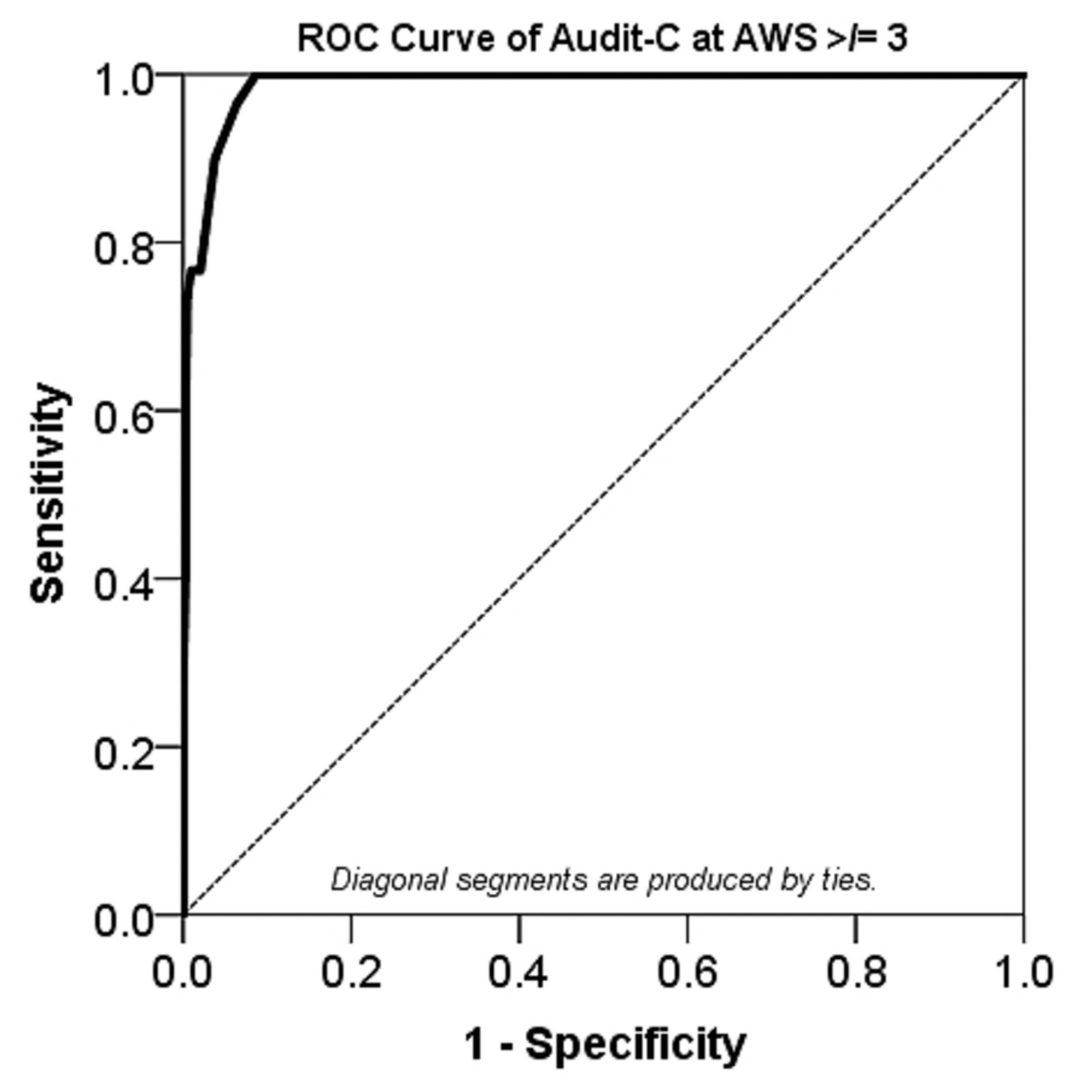
The ROC analysis of AUDIT-C scores using AWS score ≥ 3 to define alcohol withdrawal syndrome Area under the ROC = 0.989 (95% CI (0.980, 0.997), p < 0.0005); A p-value < 0.05 was considered statistically significant ROC: Receiver-operating characteristic curve, AUDIT-C: Alcohol Use Disorders Identification Test-Consumption

By defining alcohol withdrawal syndrome as an AWS score of ≥ 3, an ROC analysis revealed that AUDIT-C scores of 5 or greater were predictive of AWS development (Figure 2). The area under the ROC curve was 0.989 (95% CI (0.980, 0.997), p < 0.0005), which is considered outstanding discrimination according to Hosmer et al. [31]. 

A binomial logistic regression was performed to ascertain the predictive value of the AUDIT-C score on the likelihood of a patient developing AWS. Linearity of the AUDIT-C variable with respect to the logit of the dependent variable was assessed via the Box-Tidwell (1962) procedure [32]. Based on this assessment, the AUDIT-C variable was found to be linearly related to the logit of the dependent variable (p = 0.549). Nine standardized residuals with a value > 2.600 standard deviations were identified and retained in the analysis. The logistic regression model was statistically significant, χ2(1) = 172.371, P < 0.0005. The model explained 74.3% (Nagelkerke R2) of the variance for AWS greater than or equal to three and correctly classified 98.3% of cases. The AUDIT-C was a statistically significant predictor (p < 0.0005) for AWS. For every unit increase in AUDIT-C score, the odds of having AWS increase by a factor of 2.4. For AWS, defined as an AWS score ≥ 3, an Audit-C score ≥ 5 yields a 90.0% sensitivity, a 96.2% specificity, a positive predictive value of 52.9%, and a negative predictive value of 99.5%, using the observed prevalence of 4.5% (Table 4). 

**Table 4:** Using an AUDIT-C score ≥ 5 to predict AWS AUDIT-C: Alcohol Use Disorders Identification Test-Consumption, AWS: Alcohol withdrawal syndrome

AWS defined as an AWS score ≥ 3		Estimated value	95% confidence interval
				Prevalence		4.5%	3.1% – 6.5%
Sensitivity		90.0%	72.3% – 97.4%
Specificity		96.2%	94.3% – 97.5%
Positive predictive value (PPV)		52.9%	38.6% – 66.8%
Negative predictive value (NPV)		99.5%	98.4% – 99.9%
Likelihood ratio (LR), if AUDIT-C score	≥ 5	1.13	0.76 – 1.66
	< 5	0.005	0.0016 – 0.015
Note: Likelihood ratios weighted by prevalence			

**Figure 3:**
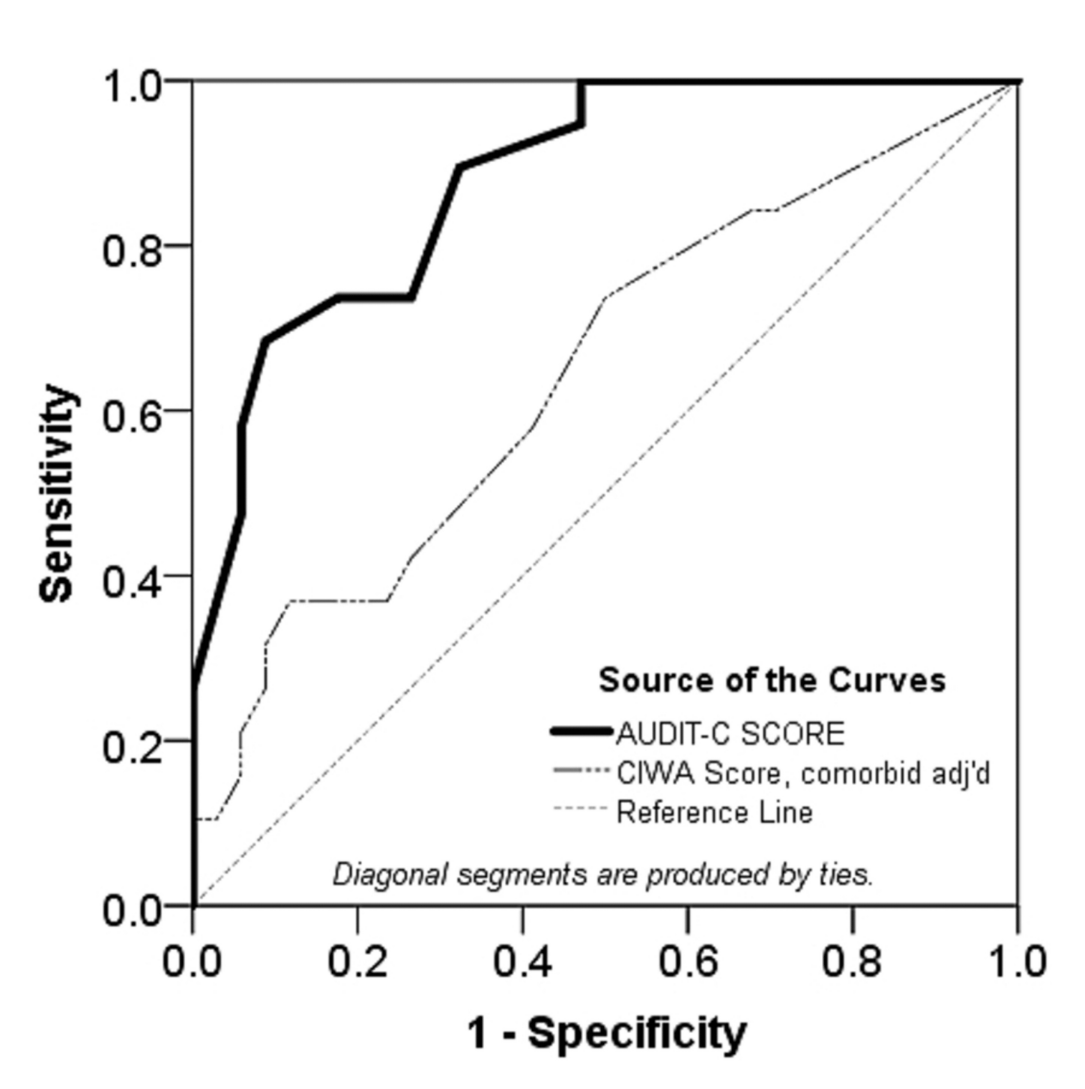
ROC analysis of AUDIT-C score vs CIWA-Ar score in predicting AWS as defined by AWS score ≥ 3 Note: The sample size for this analysis of the CIWA-Ar score is 53; Audit-C:  0.881 (95% CI (0.791, 0.971), p < 0.0005); CIWA-Ar adjusted for comorbidities: 0.646 (95% CI (0.488, 0.803), p = 0.081); A p-value < 0.05. was considered statistically significant ROC: Receiver-operating characteristic curve, AUDIT-C: Alcohol Use Disorders Identification Test-Consumption, AWS: Alcohol withdrawal syndrome

Interestingly, when CIWA-Ar scores were compared with AUDIT-C scores after adjusting for patient comorbidities, the AUDIT-C assessment outperformed CIWA-Ar in predicting AWS (Figure 3). 

In fact, adjusted CIWA-Ar scores and AUDIT-C scores were negatively correlated, Kendall's tau b = -0.203, p = 0.049. Furthermore, our data revealed that length of stay (LOS) was not statistically associated with or affected by AUDIT-C (Spearman's rho = -.023, p = 0.562), CIWA-Ar (Spearman's rho = 0.199, p = 0.153), or adjusted CIWA-Ar (Spearman's rho = 0.179, p = 0.199); and it was only very mildly correlated with the study AWS score (Spearman's rho = 0.143, p < 0.0005). The overall mean (range) LOS was 5.0 days (two to 40), and the mean (range) LOS for the subgroup with an AUDIT-C ≥ 5 was 4.6 days (two to 12). 

To provide clinicians a guide for more objective utilization of alcohol withdrawal protocols, an AUDIT-C threshold of 5 was identified by combining the binary logistic regression and ROC analyses results. As hypothesized, 48 patients (7.3%) were suboptimally managed regarding AWS and use of the CIWA-Ar protocol when this AUDIT-C threshold score was applied. Of those with AUDIT-C scores ≥ 5 (n = 51), 23 (45%) patients were not placed on CIWA-Ar for AWS prevention. This group represents the number of those in our study at risk for under/delayed treatment for AWS. Conversely, of the patients with an AUDIT-C score < 5 (n = 611), 25 patients (4.1%) were placed on CIWA-Ar without harboring a history of moderate alcohol use. This group represents the number of patients in our study at risk for over-sedation or overtreatment due to inappropriate CIWA-Ar use.

## Discussion

As a community institution that is witnessing an increase in geriatric trauma admissions, along with rising alcohol use in the general population, it is important to acknowledge that the reported prevalence of AUD amongst geriatric emergency room evaluations is roughly 10% to 15% [8,16]. Our data were consistent with the reported range of AUD prevalence, as 12% of the study population was found to have moderate alcohol use (n = 78).

Though 78 patients were identified as at risk, only 30 patients were recognized to have undergone AWS, defined as an AWS score of ≥ 3. However, 51 patients scored ≥ 5 on the AUDIT-C scale. As 51 patients at risk for AWS were identified, but only 30 experienced it clinically, this may suggest that an AUDIT-C score of ≥ 5 is too conservative. The ROC and visual cluster analysis were used in deciding on an AUDIT-C cutoff value that balanced optimization of sensitivity and specificity while prioritizing a higher false positive rate over a false negative rate. An increase in the false negative rate would have equated to missed or delayed diagnosis of AWS, which, per the literature, has been associated with increased LOS, higher costs, and higher morbidity and mortality rates [1,6,7].

Of the 25 patients who had AUDIT-C scores < 5 but were placed on the CIWA-Ar protocol, only one patient appeared to have developed AWS and required benzodiazepine treatment, with no significant effect on this patient’s length of stay, overall health, or medical care. In fact, as detailed in the results section, our data revealed that length of stay was not statistically associated with or affected by AUDIT-C, CIWA-Ar, or adjusted CIWA-Ar; however, LOS did show a very mild statistically significant correlation with the study AWS score.

Due to the limited population that experienced AWS, it is difficult to analyze this subgroup for statistically significant complications. For example, of the 23 patients with an AUDIT-C score ≥ 5 who were not placed on CIWA-Ar, two patients received benzodiazepines for agitation (based on clinician judgement). One patient had a history of recent heavy alcohol use, but the other had no documented alcohol history. Subsequent research will be focused on including patients with moderate or heavy alcohol use and excluding patients who do not drink alcohol regularly, as their risk for AWS is low. Another way to improve upon this research is to exclude patients with severe traumatic brain injuries. This injury complex may confound results, as this patient population has been observed clinically at this institution to require benzodiazepines and restraints due to their increased agitation and confusion.

There are several limitations of this study. First, although our study included a large patient population of 662 patients, most patients had an admission AUDIT-C score of 0 and had zero risk factors for the development of AWS (n = 495). These patients outnumbered the population of patients who had significant AUDIT-C scores and were at risk for developing AWS (n = 51) (Table 2). Though the proportion of patients that developed AWS in our study was comparable to the rates reported in current literature, this study was limited to only a single year of data. In future research endeavors, we intend to increase study duration to capture a larger study population and, in turn, increase the potential number of patients that would develop AWS.

The diagnosis of AWS was inconsistently documented in the EMR, making it difficult to retrospectively decide who experienced AWS. This may have contributed to the finding of 24 patients having had an AUDIT-C score > 5, yet their AWS score did not support a determination of alcohol withdrawal. As previously described, we developed an AWS score to define AWS for this study. In subsequent studies, AWS will be defined even more clearly with the inclusion of AWS-related diagnosis codes. Lastly, since the AUDIT-C questionnaire is a survey, it requires honest and accurate self-reporting by patients regarding their alcohol use. Although most patients appeared to have answered honestly, as later reflected by their development (or lack) of AWS, several patients with an AUDIT-C score of 0 (n = 10) had CIWA-Ar scores reflective of AWS and were placed on the CIWA-Ar protocol, suggestive of incongruity with self-reporting. This can improperly categorize patients and may contribute to the decreased sensitivity seen in this study. 

## Conclusions

The statistically significant and clinically meaningful findings of this study support the assertion that an AUDIT-C score of 5 or greater is predictive of AWS development in hospitalized trauma patients with a history of moderate alcohol use. Though we have provided evidence of its significance, this tool is not without flaws. Since our study population was primarily geriatric, further research is needed to determine if these findings are truly generalizable to non-geriatric trauma patients. For this reason, we encourage a combination of AUDIT-C and clinical assessment when making decisions regarding the use of withdrawal protocols and STT.

## Appendices

Appendix A

The AUDIT and AUDIT-C are evidence-based screening tools for hazardous alcohol use (Tables 6-7).

**Table 5:** The AUDIT scoring system The AUDIT is available for use in the public domain. Refer to reference [18]. AUDIT: Alcohol Use Disorders Identification Test

Questions				Scoring system						
				0	1		2	3	4	
AUDIT-C: Amount of alcohol drinking (in the past year)										
Q1.	How often do you have a drink that contains alcohol?			Never	Monthly or less		2-4 times per month	2-3 times per week	4+ times weekly	
Q2.	How many standard alcoholic drinks do you have on a typical day when you are drinking?			1-2	3-4		5-6	7-8	10+	
Q3.	How often do you have 6 or more standard drinks on one occasion?			Never	Less than monthly		Monthly	Weekly	Daily or almost daily	
AUDIT-D: Alcohol dependence										
Q4.		How often during this last year have you found that you were not able to stop drinking once you had started?		Never	Less than monthly		Monthly	Weekly	Daily or almost daily	
Q5.		How often in the last year have you failed to do what was expected of you because of drinking?		Never	Less than monthly		Monthly	Weekly	Daily or almost daily	
Q6.		How often in the last year have you needed an alcoholic drink in the morning to get you going?		Never	Less than monthly		Monthly	Weekly	Daily or almost daily	
AUDIT-P: Problem causing										
Q7.	How often during the last year have you had a feeling of guilt or remorse after drinking?			Never	Less than monthly		Monthly	Weekly	Daily or almost daily	
Q8.	How often during the last year have you been unable to remember what happened the night before because you had been drinking?			Never	Less than monthly		Monthly	Weekly	Daily or almost daily	
Q9.	Have you or someone else been injured because of your drinking?			No	---		Yes, but not in the last year	---	Yes, during the last year	
Q10.	Has a relative/friend/doctor, or another health worker been concerned about your drinking or suggested you cut down?			No	---		Yes, but not in the last year	---	Yes, during the last year	
AUDIT Scoring (C+D+P) = (0-40):										
0-7 = sensible drinking			8-15 = hazardous drinking			20+ = possible dependence				
			16-19 = harmful drinking							

**Table 6:** The AUDIT-C test The AUDIT-C is available for use in the public domain. Refer to reference [20]. AUDIT-C: Alcohol Use Disorders Identification Test-Consumption

Questions			Scoring system				
			0	1	2	3	4
AUDIT-C: Amount of alcohol drinking (in the past year)							
Q1.	How often do you have a drink that contains alcohol?		Never	Monthly or less	2 – 4 times per month	2 – 3 times per week	4+ times weekly
Q2.	How many standard alcoholic drinks do you have on a typical day when you are drinking?		1 – 2	3 – 4	5 – 6	7 – 8	10+
Q3.	How often do you have 6 or more standard drinks on one occasion?		Never	Less than monthly	Monthly	Weekly	Daily or almost daily
AUDIT-C scoring (0 – 12):							
The sum of the scores of the three questions yields the total AUDIT-C score							
A total AUDIT-C score ≥ 3 (women) and ≥ 4 (men) is suggestive of hazardous drinking or active alcohol use disorder (AUD)							
Notes:							
A.		Generally, the higher the total score, the more likely it is that a person's drinking is affecting his or her safety.					
B.		If all points are from Question 1, assume the patient is drinking below recommended limits, and the medical provider should review the patient’s alcohol intake during the past few months.					
C.		Total scores less than three are consistent with normal alcohol consumption.					

Appendix B

The CIWA-Ar score was used as an AWS score component since it is reflective of complex alcohol withdrawal (Table 8).

**Table 7:** The CIWA-Ar CIWA-Ar is not copyrighted and may be reproduced freely. Refer to references [12,13]. CIWA-Ar: Clinical Institute Withdrawal Assessment for Alcohol Revised

Symptom	Ask patient	Surveyor instruction	Score	Description of score
Agitation	Not applicable.	Observe.	0	Normal activity
			1	Slightly more activity than normal
			2-3	—
			4	Moderately fidgety/restless
			5-6	—
			7	Constant pacing or thrashing
Anxiety	“Do you feel nervous?”	Observe.	0	No anxiety, at ease
			1	Mildly anxious
			2-3	—
			4	Moderately anxious/guarded
			5-6	—
			7	Acute panic state (as in delirium or schizophrenia)
Auditory disturbances	“Are sounds harsh, frightening, or unreal?”	Observe.	0	None
			1	Very mild harshness or frightening quality
			2	Mild
			3	Moderate
			4	Moderately severe hallucinations
			5	Severe hallucinations
			6	Extremely severe hallucinations
			7	Continuous hallucinations
Headache/fullness in the head	“Does your head feel different, like a band around it?”	Not applicable.	0	None
			1	Very mild
			2	Mild
			3	Moderate
			4	Moderately severe
			5	Severe
			6	Very severe
			7	Extremely severe
Nausea and vomiting	“Do you feel sick to your stomach?"	Observe.	0	No nausea or vomiting
			1	Mild nausea, no vomiting
			2–3	—
	"Have you vomited?”		4	Intermittent nausea with dry heaves
			5–6	—
			7	Constant nausea, frequent dry heaves and vomiting
Orientation and sensorium		Not applicable.	0	Oriented, can do serial additions
	“What day is it?		1	Fails serial additions or uncertain of date
	"Where are you?"		2	Disoriented by ≤2 days
	"Who am I?”		3	Disoriented by >2 days
			4	Disoriented for place/person
Paroxysmal sweats	Not applicable	Observe	0	No sweating
			1	Barely perceptible, palms moist
			2-3	—
			4	Beads of sweat on the forehead
			5-6	—
			7	Drenching sweats
Tactile disturbances	“Any itching, pins and needles, burning, numbness, or feeling of bugs crawling?”	Observe.	0	None
			1	Very mild itching, pins/needles, burning, or numbness
			2	Mild sensations
			3	Moderate sensations
			4	Moderately severe hallucinations
			5	Severe hallucinations
			6	Extremely severe hallucinations
			7	Continuous hallucinations
Tremor	Ask patient to extend arms, fingers spread.	Observe	0	No tremor
			1	Not visible, but palpable fingertip to fingertip
			2–3	—
			4	Moderate, with arms extended
			5–6	—
			7	Severe, even without arm extension
Visual disturbances	“Is light too bright, colors different, eyes hurting, or seeing unreal things?”	Observe	0	None
			1	Very mild sensitivity
			2	Mild
			3	Moderate
			4	Moderately severe hallucinations
			5	Severe hallucinations
			6	Extremely severe hallucinations
			7	Continuous hallucinations
CIWA-Ar score calculation			CIWA-Ar Score Interpretation	
Sum all the symptom domain scores to produce a single total score for the CIWA-Ar			Total Score	Interpretation
			0 to 9	Very mild withdrawal
			10 to 15	Mild withdrawal
			16 to 20	Modest withdrawal
			21 to 67	Severe withdrawal
